# Supplementary material for: Changes in the Gut Microbiota Composition during Implantation of Composite Scaffolds Based on Poly(3-hydroxybutyrate) and Alginate on the Large-Intestine Wall
Source: Polymers (Basel). 2023 Sep 4;15(17):3649. doi: 10.3390/polym15173649 (PMC10489921; doi:10.3390/polym15173649)
Supplement: Supplementary file 1 [file polymers-15-03649-s001.zip › polymers-2552418-supplementary.pdf]

Supplementary Materials

Changes in the Gut Microbiota Composition during Implantation of Composite Scaffolds Based on Poly(3-Hydroxybutyrate) and Alginate on the Large-Intestine Wall

Andrei A. Dudun [1], Dariana V. Chesnokova [2], Vera V. Voinova [2], Anton P. Bonartsev [2] and Garina A. Bonartseva [1,*]

[1] A.N. Bach Institute of Biochemistry, Research Center of Biotechnology of the Russian Academy of Sciences, Leninsky Ave, 33, Bld. 2, 119071 Moscow, Russia; dudunandrey@mail.ru (A.A.D.); bonar@inbi.ras.ru (G.A.B.)

[2] Faculty of Biology, M.V. Lomonosov Moscow State University, Leninskie Gory 1-12, 119234 Moscow, Russia; daryana8@yandex.ru (D.V.C); veravoinova@mail.ru (V.V.V.); ant_bonar@mail.ru (A.P.B.)

* Correspondence: bonar@inbi.ras.ru; Tel.: +7(495) 954-40-08

Cultivation of probiotic bacteria

Bifidobacterium longum MC-42 and *Lactobacillus fermentum* 90 TS-4 were used in the studies. Both strains were cultured in liquid MRS medium (Sigma-Aldrich, Darmstadt, Germany) at 25°C and pH 5.7 ± 0.2. The media were autoclaved at 118°C for 15 minutes. Bacteria were cultured on a New Brunswick Scientific classic series microbiological rocker (New Brunswick Scientific Co., Inc, Edison, NJ, USA) at 37°C (*B. longum* - without agitation, *L. fermentum* - with agitation at 120 rpm) for 4-6 h until the bacterial suspensions reached optical density values of 0.8-1. The density of the suspensions was determined by the turbodimetric method on the spectrophotometer SF-2000 (OKB-Spectr, Saint Petersburg, Russia) at wavelength λ = 600 nm and optical path length of 1 cm. For correct analysis, samples with optical density values higher than 0.8 units were diluted with potassium phosphate buffer (Sigma-Aldrich, Darmstadt, Germany) the required number of times.

Surgeries

After induction of anaesthesia, Wistar rats underwent laparotomy and withdrawal of the large intestine from the abdominal cavity with fixation of a section of this intestine using two Kocher clamps. In the early stages of the study, the large intestine was subjected to traumatisation and ischemia by contact with the metal parts of the clamps, so the clamps themselves were wrapped with hygroscopic medical cotton wool beforehand to prevent damage to the serosal muscular tissue of the intestine during direct grasping (Figure S1B). Damage to the large intestine was performed with surgical scissors centrally between two 3–4 mm wide Kocher clamps (Figure S1C,D). A series of surgeries were then performed on the rats at the site of the lesion with an intestinal patch 1.5–2 cm long and 0.5 cm wide (Figure S1E). A modified Lambert suture was used to close the damaged area in the large intestine: the use of a standard suture was problematic due to the fact that it only captures the serosal-muscular (outer case) tissue of the intestine, so a modified "through" Lambert suture was used [21].

The colon was sutured strictly perpendicularly. To prevent drying of the GI tract, the operation site was periodically moistened with physiological solution (0.9% NaCl) with the antiseptic Furacilin 0.4 g/L. To achieve

tightness, the frequency of Lambert sutures should be at least 3 stitches per 1 cm of length both lengthwise and transversely at the site of the large bowel injury (Figure S1F, G) [22]. During the technique for the damaged colon, the suture frequency for implantation of the PHB/ALG patch required standard 3 by 3 sutures (Figure S1H) [22,57]. At the end of surgery, the intestinal patch was fixed with marginal sutures to the wall of the large intestine, and the abdominal wall was closed with transverse sutures at a frequency of 2 stitches per 1 cm of length and treated with the antibacterial drug Streptocide [23].

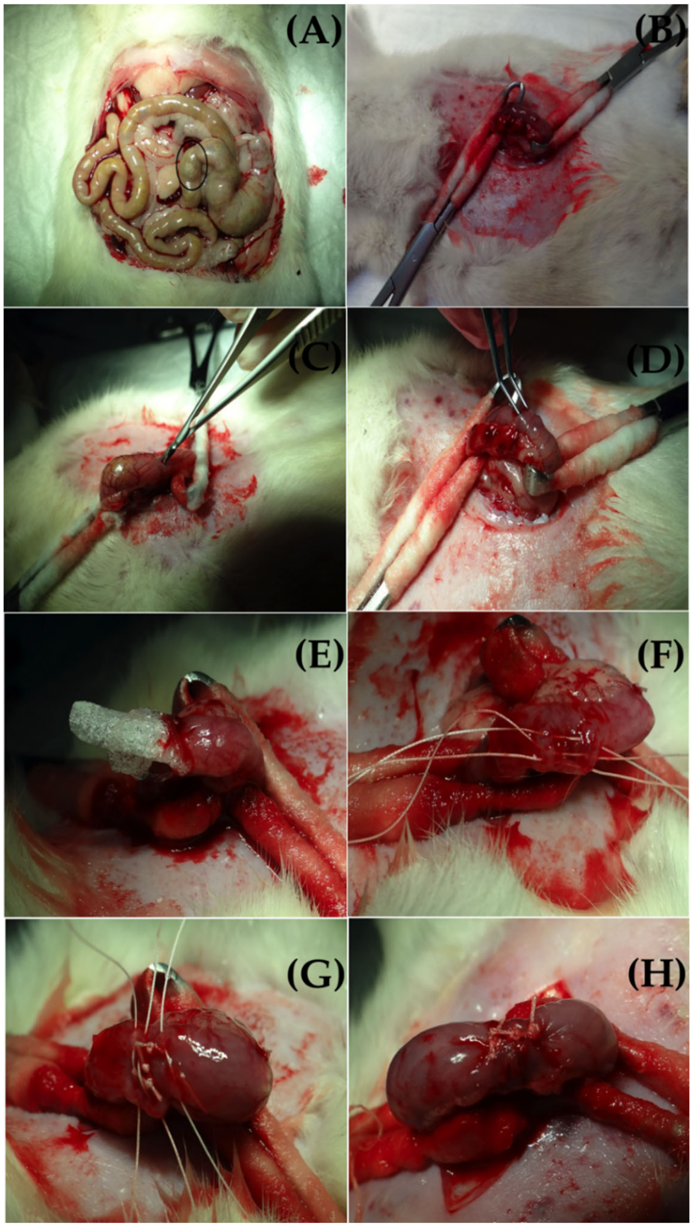

Figure S1. Surgeries for implantation of different variants of PHB/ALG biopolymer scaffolds. (**A**)—Location of the defect (area highlighted with a black circle) and subsequent implantation of polymeric PHB/ALG scaffolds into the large intestine, which exits directly from the cecum. (**B**)—Fixation of the large intestine with Kocher clamps. (**C**)—Creation of a mechanical defect of the large intestine in rats using surgical scissors. (**D**)—Performed incision between two Kocher clamps. (**E**)—Implantation of an intestinal biopolymer scaffold in the form of a PHB/ALG patch. (**F**)—Lambert's longitudinal sutures. (**G**)—Lambert's transverse sutures. (**H**)—Implanted patch with 3 × 3 sutures.

References

21. Burch, J.M.; Franciose, R.J.; Moore, E.E.; Biffl, W.L.; Offner, P.J. Single-Layer Continuous versus Two-Layer Interrupted Intestinal Anastomosis: A Prospective Randomized Trial. *Ann. Surg.* **2000**, *231*, 832–837. https://doi.org/10.1097/00000658-200006000-00007.
22. Nickkholgh, A.; Contin, P.; Abu-Elmagd, K.; Golriz, M.; Gotthardt, D.; Morath, C.; Schemmer, P.; Mehrabi, A. Intestinal transplantation: review of operative techniques. *Clin. Transplant.* **2013**, *27*, 56–65, https://doi.org/10.1111/ctr.12190.
23. Bosmans, J.W.A.M.; Jongen, A.C.H.M.; Boonen, B.T.C.; van Rijn, S.; Scognamiglio, F.; Stucchi, L.; Gijbels, M.J.J.; Marsich, E.; Bouvy, N.D. Comparison of three different application routes of butyrate to improve colonic anastomotic strength in rats. *Int. J. Color. Dis.* **2016**, *32*, 305–313, https://doi.org/10.1007/s00384-016-2718-z.
57. Weber, C. Commensal bacteria and intestinal surgery complications. *Nat. Rev. Gastroenterol. Hepatol.* **2015**, *12*, 371–371, https://doi.org/10.1038/nrgastro.2015.92.
